# Probing the proteome

**DOI:** 10.7554/eLife.110102

**Published:** 2026-01-07

**Authors:** Wei-Hsiang Lin, Chia-Liang Cheng

**Affiliations:** 1 Institute of Molecular Biology, Academia Sinica Taipei Taiwan; 2 Department of Physics, National Dong Hwa University Hualien Taiwan

**Keywords:** Raman spectroscopy, proteome, stoichiometry conservation, low dimensionality, *M. tuberculosis*, *M. bovis*, *E. coli*, Human, *S. cerevisiae*, *S. pombe*, Other

## Abstract

Raman spectroscopy can be used to predict cellular physiology and proteome composition in *E. coli*.

**Related research article** Kamei KF, Kobayashi-Kirschvink KJ, Nozoe T, Nakaoka H, Umetani M, Wakamoto Y. 2025. Revealing global stoichiometry conservation architecture in cells from Raman spectral patterns. *eLife* **14**:RP101485. doi: 10.7554/eLife.101485.

When a photon of light is scattered by a molecule, the energy of the photon generally remains the same. In a small number of cases, however, the energy of the photon can change. This process is called Raman scattering and was discovered in 1928 by the Indian physicist CV Raman, who went on to receive the Nobel Prize in Physics two years later for discovering it.

Raman scattering typically happens when the photon excites the molecule from its initial state into a state with a higher vibrational energy, which results in the photon having less energy (and hence a longer wavelength) than it had before. Less frequently, the scattered photon can have more energy (and hence a shorter wavelength) than it had initially. Every molecule has a unique set of energy states, so analyzing the Raman spectrum – that is, measuring the number of the scattered photons as a function of wavelength – can tell us quite a lot about the molecule itself. Moreover, since the energy levels of the molecule are influenced by other molecules in the vicinity, the Raman spectrum also contains information about the surrounding environment.

Historically, Raman spectroscopy has been a popular tool for studying molecules in chemistry, and also for studying lattice vibrations in solid materials in physics. Now, thanks to the fact that it is a non-contact and non-invasive technique, it is emerging as a potentially powerful technique in the life and biomedical sciences (Chandra et al., 2024; Hose et al., 2024). For cells and tissues, the Raman spectrum contains information about the different kinds of molecules they contain, including metabolites, sugars, RNA molecules, proteins and the extracellular matrix (Figure 1). This means that Raman spectroscopy can be used to detect various chemical components in cells, and to distinguish between cells growing in different physiological conditions (Devitt et al., 2018). Furthermore, modern technological advances make it possible to measure the Raman spectrum at multiple positions in a sample: this approach, which is called Raman mapping, provides information on the spatial distribution of different molecules within the sample (Butler et al., 2016).

**Figure 1.**
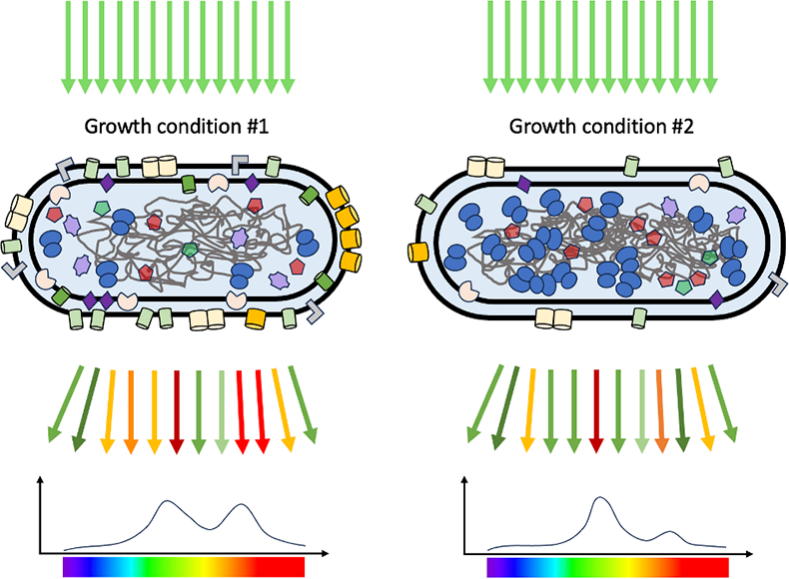
Raman spectra and proteome composition. Schematic representation showing how Raman spectroscopy can be used to study the proteome of *E. coli* under different growth conditions. Light of a single wavelength (532 nanometers; top) is directed towards the *E. coli* (middle), and the light scattered by the various proteins in the proteome is recorded as a function of wavelength (horizontal axis, bottom). The individual proteins (represented here by different shapes and colors) all have different Raman spectra, so the overall Raman spectrum extends over a wide range of wavelengths and contains various peaks and troughs. If the growth conditions are adjusted, the composition of the proteome will also change, as will the Raman spectrum. By jointly analyzing the Raman spectra for 15 sets of growth conditions with existing data on the proteomes for these growth conditions, Kamei et al. showed that Raman data can be used to predict cellular physiology and proteome composition. In addition to proteins, other biomolecules (such as sugars) also contribute to the Raman spectra.

In biological samples, the proteome – the set of all the proteins that can be expressed by a genome – accounts for about 50% of dry weight and is therefore a major contributor to the Raman spectrum (Bray, 2001; Neidhardt et al., 1990). It is known that cells adjust the relative abundances of the various proteins in their proteome to cope with different environments: in *E. coli*, for example, the fraction of ribosomal proteins increases when conditions enable fast growth (Erickson et al., 2017). Similar results have been seen with budding yeast (Metzl-Raz et al., 2017).

In 2016, Matthias Heinemann and co-workers used mass spectrometry to measure the abundance of more than 2300 proteins in *E. coli* under 22 different sets of growth conditions (Schmidt et al., 2016). Now, in eLife, Ken-ichiro Kamei, Yuichi Wakamoto and colleagues at the University of Tokyo and other institutions in Japan and the US report that Raman spectroscopy can also be used to infer proteome compositions (Kamei et al., 2025).

The researchers recorded Raman spectra for *E. coli* under 15 of the 22 growth conditions used by the Heinemann group, and conducted a detailed statistical analysis of the correlations between the proteome and their dataset of Raman spectra. Specifically, they employed a technique called linear discriminant analysis (LDA) which highlights the directions (in 15-dimensional space) that maximize the differences between the Raman data for the 15 sets of growth conditions. These directions are called the LDA axes. Interestingly, the first LDA axis is strongly correlated with growth rate, and the other LDA axes can distinguish between stationary phase and carbon-rich conditions which support rapid growth. These results suggest that Raman spectra are closely correlated with cellular physiology.

Strikingly, Kamei et al. show that it is possible to infer proteomic composition from Raman spectroscopic data. In particular, with the LDA analysis, they identify a group of proteins that participates in replication, transcription and translation. Moreover, the proteins in this group are regulated with fixed ratios under different physiological states. Kamei et al. also found that a similar group of proteins is conserved from bacteria and yeast to humans, indicating the importance of relative abundance in protein regulation.

It is remarkable that Raman spectra can, with the help of statistical inference, be used to predict cellular physiology and proteome composition. By making this possible, the work of Kamei et al. has opened up a promising direction for probing cellular regulation and dynamics.
